# Molasses-Silver Nanoparticles: Synthesis, Optimization, Characterization, and Antibiofilm Activity

**DOI:** 10.3390/ijms231810243

**Published:** 2022-09-06

**Authors:** Rabab A. Dorgham, Mohamed N. Abd Al Moaty, Khim Phin Chong, Bassma H. Elwakil

**Affiliations:** 1 Botany and Microbiology Department, Faculty of Science, Alexandria University, Alexandria 21568, Egypt; 2 Chemistry Department, Faculty of Science, Alexandria University, Alexandria 21568, Egypt; 3 Faculty of Science and Natural Resources, Universiti Malaysia Sabah, Jalan UMS, Kota Kinabalu 88400, Sabah, Malaysia; 4 Department of Medical Laboratory Technology, Faculty of Applied Health Sciences Technology, Pharos University in Alexandria, Alexandria 21321, Egypt

**Keywords:** green silver nanoparticles, antibiofilm, response surface methodology

## Abstract

Biofilms are matrix-enclosed communities of bacteria that are highly resistant to antibiotics. Adding nanomaterials with antibacterial activity to the implant surfaces may be a great solution against biofilm formation. Due to its potent and widespread antibacterial effect, silver nanoparticles were considered the most potent agent with different biological activities. In the present investigation, silver nanoparticles (AgNPs) were newly synthesized as antibiofilm agents using sugarcane process byproduct (molasses) and named Mo-capped AgNPs. The synthesized nanoparticles showed promising antimicrobial activity against *S. aureus* ATCC 6538 and *C. albicans* DAY185. Statistically designed optimization through response surface methodology was evaluated for maximum activity and better physical characteristics, namely the nanoparticles’ size and polydispersity index (PDI), and it was revealed that molasses concentration was the main effective factor. Minimal biofilm eradication concentration (MBEC) of Mo-capped AgNPs against *S. aureus* ATCC 6538 and *C. albicans* DAY185 was 16 and 32 µg/mL, respectively. Scanning electron microscope study of Mo-capped AgNP-treated biofilm revealed that AgNPs penetrated the preformed biofilm and eradicated the microbial cells. The optimally synthesized Mo-capped AgNPs were spherically shaped, and the average size diameter ranged between 29 and 88 nm with high proportions of Ag^+^ element (78.0%) recorded. Fourier-transform infrared spectroscopy (FTIR) analysis indicated the importance of molasses ingredients in capping and stabilizing the produced silver nanoparticles.

## 1. Introduction

A surface-accreted microcolony that is encompassed by a self-produced extracellular polymeric matrix (EPS) is referred to as a microbial biofilm. This EP is a biosynthetic polymer composed of proteins, phospholipids, polysaccharides, and nucleic acids, and serves as both a binding agent and a barrier against common antibiotics and microbicidal substances. According to statistics, bacteria within biofilms might be up to 1000 times more resistant to antibiotics than their planktonic [1]. This increasing resistance to antimicrobial treatment is harmful to human health since biofilms are the cause of 80% of all bacterial infections, leading to many biofilm-related diseases becoming chronic [1].

Nanoparticles (NPs) are characterized by a large surface-to-volume ratio with high surface reactivity. NPs were used widely in chemistry, physics, and especially in biomedicine [2,3]. The use of natural systems in nanoparticles synthesis has many advantages that have drawn significant researchers’ interest during the past 10 years. Silver (Ag) and gold (Au)-NPs might be manufactured using naturally occurring reducing materials from different sources such as plants, fungus, honey, chitosan, bacteria, and yeast [4]. Silver nanoparticles have been studied as antibiofilm materials in several studies due to their potent antibacterial activities [5]. Goderecci et al. [6] evaluated the antibacterial activity of nanosilver oxide (AgO) films against *Staphylococcus aureus* and *Escherichia coli* after being deposited on titanium foil discs. Thukkaram et al. [7] reported that colony-forming units (CFUs) of *S. aureus* and *E. coli* were decreased by 4- to 5-logs using AgNP nanocomposites made of an amorphous hydrocarbon matrix and nanosilver with an average size of 25 nm. Taglietti et al. [8] studied the antibiofilm activity of an AgNP (25 nm in size) monolayer attached to an amino-silanized glass surface and discovered that *S. epidermidis* CFU was reduced by 5-logs. On the other hand, it was reported that by varying the plant concentration (reducing agent concentration), different silver nanoparticle sizes can be achieved [9].

The expansion of the world’s population has raised the amount of waste produced globally. One of the waste products from the sugarcane (*Saccharum officinarum*) processing industry is molasses. It has several health benefits, e.g., antioxidant, antiobesity, anticancer, antimicrobial, etc. [10]. One of our main goals was to use an industrial waste product, namely molasse, as a reducing and capping agent to synthesize an economically cheap silver nanoparticle.

The use of traditional methods for experimental optimization, where one factor is changed at a time while the others are fixed, has many drawbacks. These methods use many experiments to show the effects of each variable individually but ignore the effects of interactions between the various factors under study [11]. However, using statistical designs facilitate the appropriate selection of significant factors from the various process-affecting variables. It also aids in comprehending how many prominent factors interact with one another [12,13]. Response surface methodology (RSM) is a mathematical and statistical analytic technique that involves the interactions between various process factors in order to enhance and improve the process settings. RSM has been used to determine the Ideal conditions for a variety of biotechnological processes by analyzing the interactions between the process variables [14].

In the present work, molasses-capped silver nanoparticles (Mo-capped AgNPs) were newly synthesized using an industrial waste product (molasses) as a capping and reducing agent using statistical design analyses. The present research supports the development of Mo-capped AgNPs as potential antibiofilm nanosized agents to eradicate microbial biofilm.

## 2. Results and Discussion

### 2.1. Liquid Chromatography–Mass Spectroscopy (LCMS) Analysis

**Figure 1:**
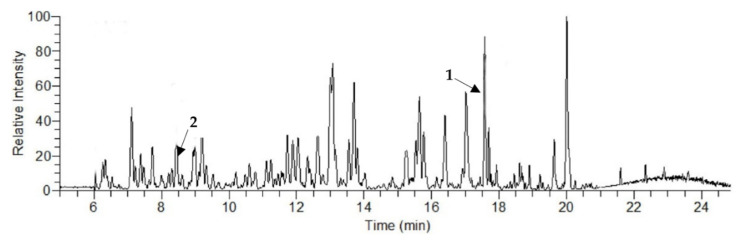
LCMS chromatogram of sugarcane molasses where (1) is diosmin and (2) is chlorogenic acid.

LCMS analysis of the obtained molasses showed a complex chromatogram with major peaks referring to eight polyphenols, and the highest concentration was noticed with diosmin (13.1 μg/g), followed by chlorogenic acid (5.91 μg/g) (Figure 1 and Table 1). Deseo et al. [15] assessed the polyphenol concentration in molasses and reported that diosmin showed the highest concentration (19.45 μg/g), followed by syringic acid (10.90 μg/g) and chlorogenic acid (6.53 μg/g), while other reported polyphenols concentrations were less than 1 μg/g.

### 2.2. Mo-Capped AgNP Synthesis

**Figure 2:**
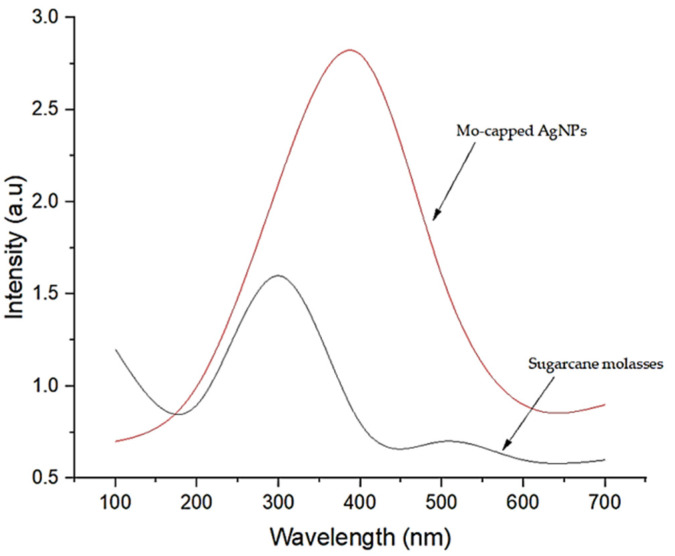
UV–vis spectroscopy of the prepared Mo-capped AgNPs.

AgNPs were successfully synthesized using molasses, which was confirmed by the presence of a major peak around 390 nm through UV–vis spectroscopy study (Figure 2). UV–vis range was dependent upon the particle type, size, and shape, while the dynamic light scattering (DLS) of the synthesized nanoparticles revealed that the nanoparticles’ size was 90.7 nm, while the polydispersity index was 0.85. Vilchis-Nestor et al. [16] synthesized silver nanoparticles by using *Camellia sinensis* extract as a reducing agent, and they indicated that the reduction of the Ag^+^ ions took place extracellularly.

Data in Table 2 reveal that Mo-capped AgNPs showed maximum activity against *S. aureus* ATCC 6538 with an inhibition zone diameter of 19.5 mm. Banerjee et al. [17] synthesized AgNPs using leaf extracts of three plants, *Musa balbisiana* (banana), *Azadirachta indica* (neem), and *Ocimum tenuiflorum* (black tulsi), and reported that AgNPs prepared from banana leaf extract had the maximum inhibition activity when tested against *Bacillus* spp., while AgNPs synthesized using neem and tulsi leaf extracts showed similar antimicrobial properties.

### 2.3. Optimization of Mo-Capped AgNPs

**Figure 3:**
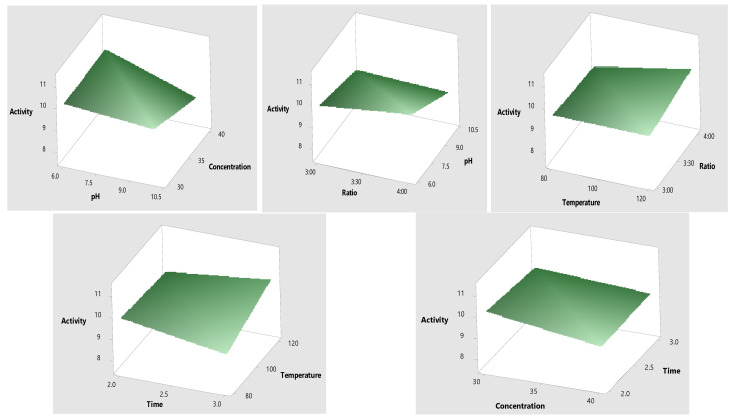
Three-dimensional surface plots of Mo-capped AgNPs.

**Figure 4:**
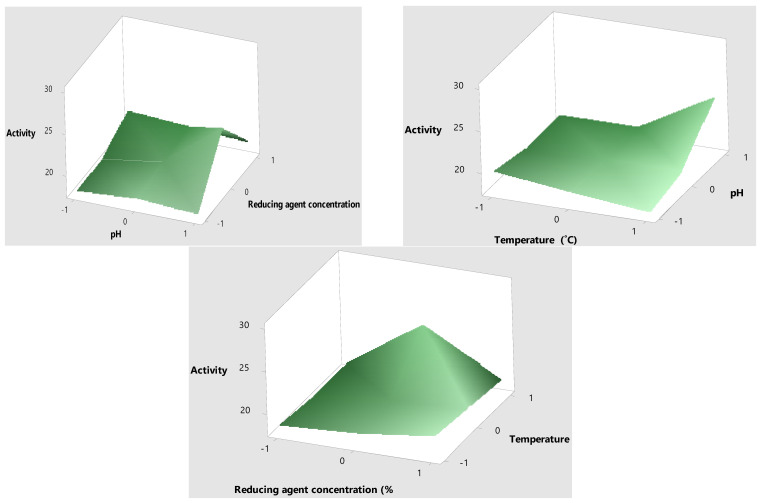
Three-dimensional response surface plot for Mo-capped AgNP synthesis.

The response surface methodology design was chosen to optimize the antimicrobial activity (R1), particle size (R2), and polydispersity index (R3). Different environmental factors were chosen, namely molasses (reducing agent), concentration (%), pH, ratio (reducing agent: AgNO_3_), reaction temperature (°C), and reaction time (h). Data in Table 3 show that the highest antimicrobial activity with the lowest PDI value and minimum particle size were obtained when the molasses concentration was 30%, the pH was 10, and the ratio of molasses–AgNO_3_ was 4:1 at 120 °C for a 3 h reaction time. SPSS© analysis revealed that molasses concentration was the main effective factor in the green synthesis of Mo-capped AgNPs (Figure 3). The results of the analysis of variance (ANOVA) are elucidated in Table S1, where the significance of each variable studied with a probability (*p*) value of less than 0.0500 indicated that model terms such as A, B, C, AB, A^2^, B^2^, and C^2^ were significant. Furthermore, the predicted coefficient of determination (Pred R^2^) and the adjusted coefficient of determination (Adj R^2^) were found to be in reasonable agreement, thus confirming the significance of the used quadratic model (Table S2). Data in Figure 4 represent the 3D response surface plot for AgNP synthesis, and it was noticed that the antimicrobial activity increased when the molasses concentration decreased. The significant factors were furtherly optimized through the Box–Behnken design to reach the maximum activity. The variables determined from the Box–Behnken design model were a molasses concentration of 30%, a pH of 10, and a temperature of 120 °C (Table 4).

Pourmortazavi et al. [18] optimized the green synthesis of AgNPs by controlling the operating factors namely: plant extract and silver ion concentrations, reaction time, and the extraction temperature. It was revealed that by increasing the plant extract concentration, raising the temperature of the extraction process, and increasing the synthesis reaction time, the size of AgNPs decreased, while by increasing the silver ion concentration in the solution the nanoparticles’ size increased. Ebrahimzadeh et al. [19] proved that by increasing the pH, the ligand protonation decreases, and the condition becomes more favorable for complex formation and sorption of metal ions to the imprinted sorbent.

### 2.4. Antimicrobial Activity of Mo-Capped AgNPs

Antimicrobial activity of Mo-capped AgNPs was assessed through minimum inhibitory concentration (MIC), microbial lethality curve, and transmission electron microscopy (TEM). Data in Table 5 show that the MIC values of the synthesized Mo-capped AgNPs were 4 and 16 μg/mL against *S. aureus* ATCC 6538 and *C. albicans* DAY185, respectively. Concerning MIC and MBC values, it was concluded that Mo-capped AgNPs had bactericidal and fungicidal effects with an MIC index ≤ 4.

**Figure 5:**
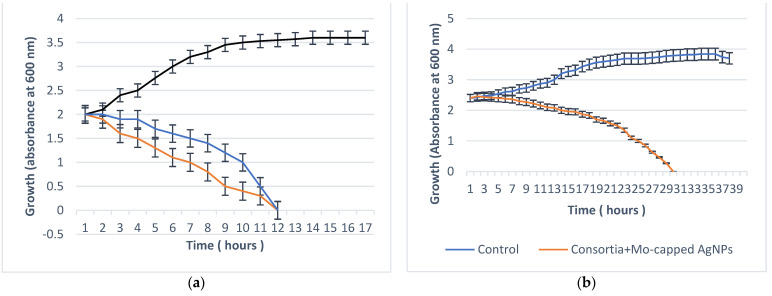
Microbial lethality curve against *S. aureus* ATCC 6538 (red line) and *C. albicans* DAY185 (blue line) (**a**) and consortia of both (**b**).

*S. aureus* ATCC 6538 and *C. albicans* DAY185 cells were subjected to MIC values at different time intervals, and the microbial lethality curve was evaluated. Data in Figure 5 show that the microbial growth of *C. albicans* DAY185 reached 0 after 12 h incubation with Mo-capped AgNPs. On the other hand, the synthesized Mo-capped AgNPs successfully inhibited the growth of microbial consortia (*S. aureus* ATCC 6538 + *C. albicans* DAY185) after 30 h.

**Figure 6:**
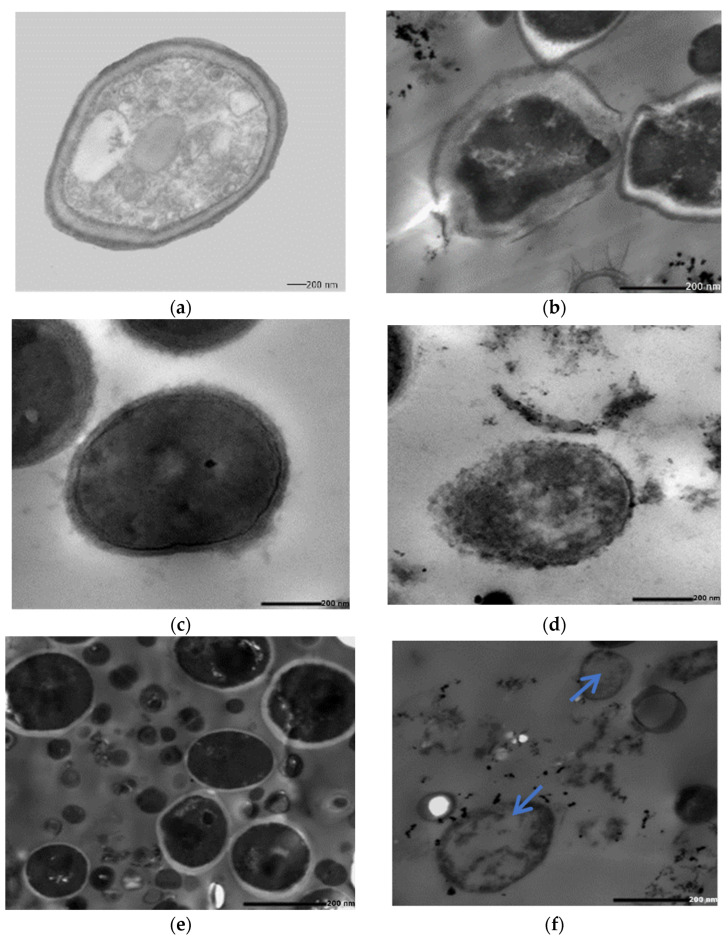
Transmission electron microscope study of *C. albicans* DAY185 control (**a**), *C. albicans* DAY185 treated with Mo-capped AgNPs (**b**), *S. aureus* ATCC 6538 control (**c**), *S. aureus* ATCC 6538 treated with Mo-capped AgNPs (**d**), consortia control (**e**), and consortia treated with Mo-capped AgNPs (**f**). Blue arrow: ghost cells.

TEM study was applied to Mo-capped AgNP-treated cells of *S. aureus* ATCC 6538 and *C. albicans* DAY185 and consortia of both. The results presented in Figure 6 indicate that AgNPs were adsorbed to the cell surface, followed by cell penetration and interaction with the intracellular components, and the cells turned into ghost cells.

Nishio et al. [20] stated that the tested Gram-positive bacteria were more sensitive than Gram-negative bacteria, and when treated with AgNPs with *S. aureus*, they showed the lowest MIC values. Haque et al. [21] stated the antimicrobial activities of AgNPs and declared that at 40 mg/mL AgNPs were able to suppress the growth of *E. coli* completely after 12 h. Morones et al. [22] revealed that AgNPs antimicrobial activity was due to their extremely large surface area, which provides better contact with microorganisms’ cell surfaces. AgNPs get attached to the cell wall and penetrate the bacterial cell to interact with sulfur-containing proteins and phosphorus-containing compounds (e.g., DNA). AgNPs preferably attack the respiratory chain and disturb the cell division, finally leading to cell death. AgNPs also release silver ions in bacterial cells, which enhance their bactericidal activity. Similarly, Ag/Bi_2_MoO_6_ (Ag/BMO) nanozyme was synthesized by Cao and coworkers [23] through solvothermal reaction and photoreduction, which showed bactericidal effects against methicillin-resistant *S. aureus* (MRSA). They explained that the observed effect was due to the corporation of peroxidase-like activity, NIR-II photodynamic behavior, and acidity-enhanced release of Ag^+^.

### 2.5. Antibiofilm Activity

#### 2.5.1. Minimal Biofilm Eradication Concentration (MBEC)

Antibiofilm activity of the newly synthesized AgNPs was assessed by several techniques, namely MBEC, scanning electron microscopy (SEM), and antibiofilm formation effect. Data in Table 6 reveal that MBEC of Mo-capped AgNPs against *S. aureus* ATCC 6538 and *C. albicans* DAY185 were 16 and 32 µg/mL, respectively. Palanisamy et al. [24] concluded that the uptake of the AgNPs could be remarkably reduced as the rate of biofilm formation increased.

#### 2.5.2. SEM of the Treated Biofilm

**Figure 7:**
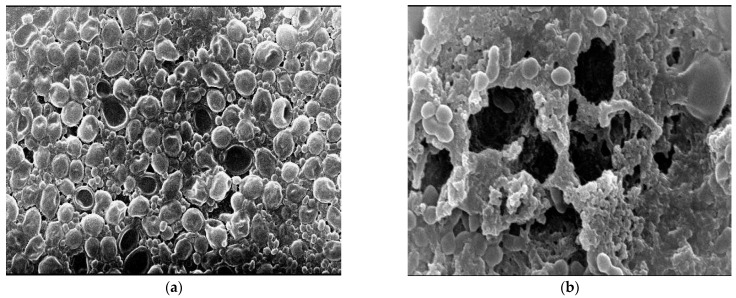
AgNP-treated biofilm: control (×5000) (**a**) and Mo-capped AgNP-treated biofilm (×15,000) (**b**).

*S. aureus* ATCC 6538 and *C. albicans* DAY185 polymicrobial cultures (consortia) grown without AgNPs exhibited the expected normal cellular morphology with smooth cell surfaces, and the microbial cells were arranged with noticed exopolysaccharides matrix (Figure 7a). Under the same growth conditions but in the presence of Mo-capped AgNPs (32 and 64 μg/mL, respectively), *S. aureus* ATCC 6538 and *C. albicans* DAY185 biofilm showed dramatically restricted bacterial colonization, the biofilm formation was very much patchy with observed changes in the cellular morphology, and the microbial colonization was inhibited (Figure 7b). It was demonstrated that AgNPs penetrated the preformed biofilm and eradicated the microbial cells. Degradation of established biofilms occurs either by the killing of formed bacteria or the detachment of living ones. Biofilm formation plays an essential role in invading the host immune defenses and increasing the antibiotic resistance, which helps in the persistence of microbial infections [24]. The obtained results agreed with Ansari et al. [25], who revealed in the presence of AgNPs, the cell morphology of *E. coli* changed, and the bacterial colonization on the surfaces was inhibited.

### 2.6. Characterization of AgNPs

**Figure 8:**
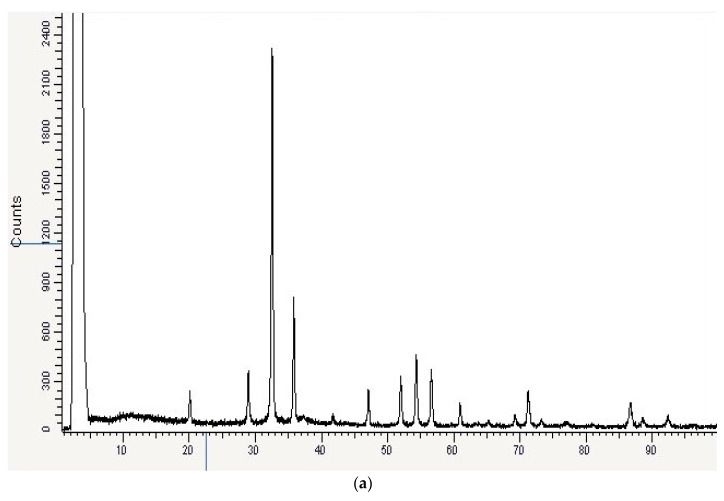

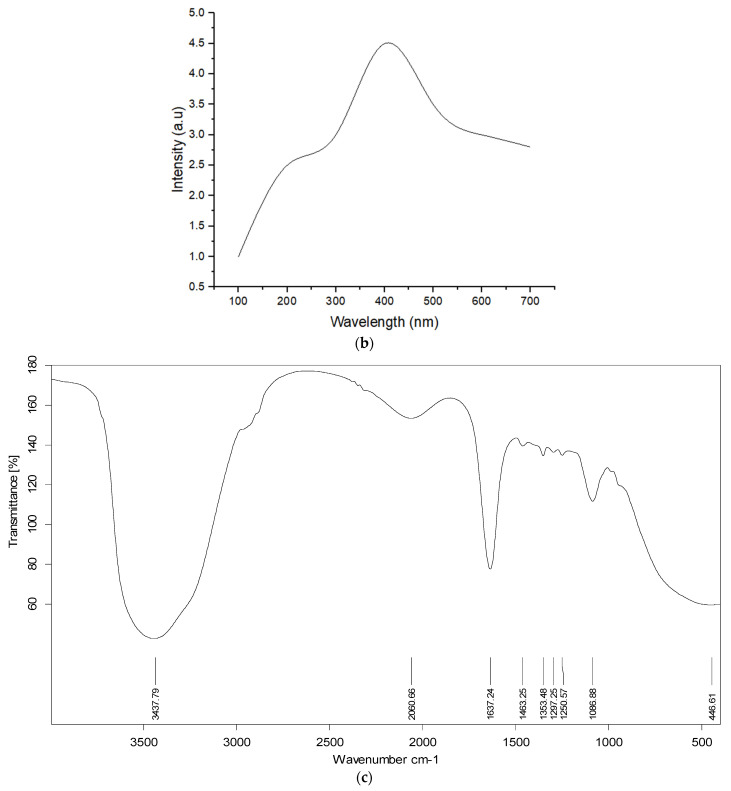

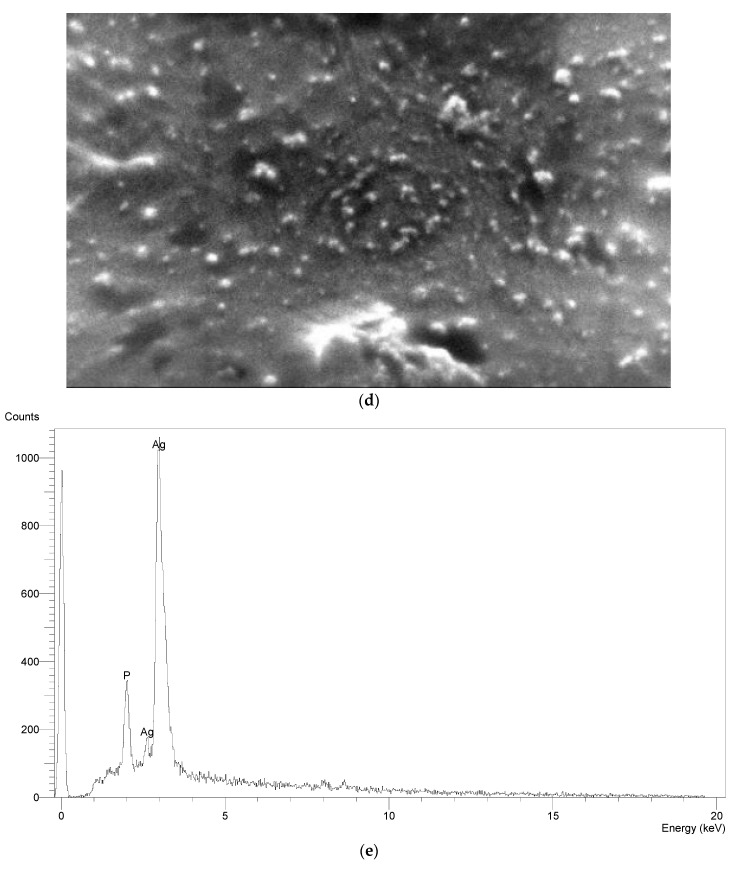

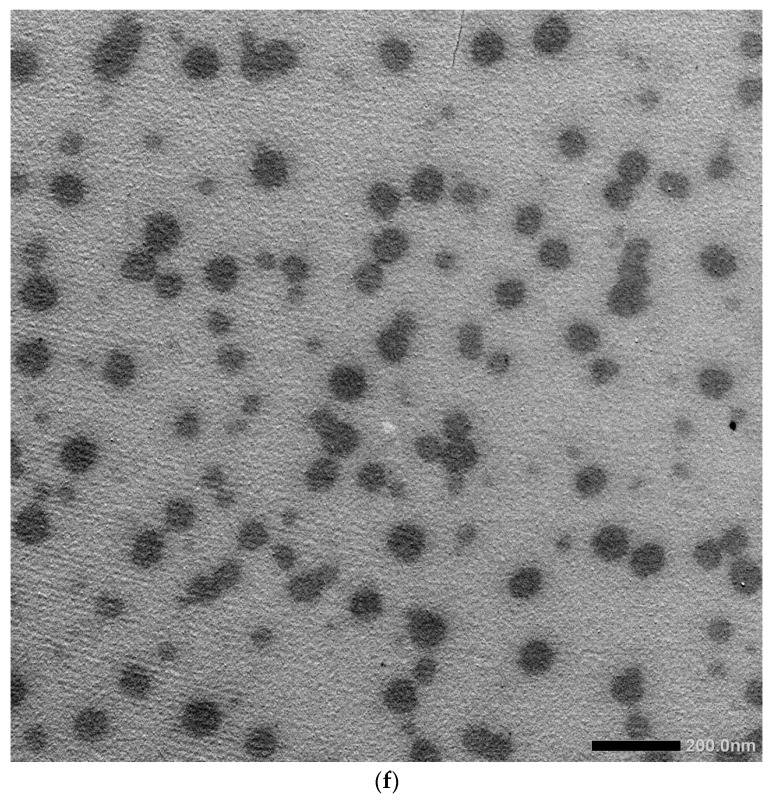
Mo-capped AgNPs characterization using X-ray diffraction (**a**), UV–vis (**b**), Fourier-transform infrared spectroscopy (**c**), scanning electron microscopy (×15,000) (**d**), energy-dispersive X-ray (**e**), and transmission electron microscope (**f**) analyses.

The optimized Mo-capped AgNPs was characterized through ultraviolet–visible (UV–vis), scanning electron microscopy (SEM), energy-dispersive X-ray (EDX), Fourier-transform infrared spectroscopy (FTIR), transmission electron microscopy (TEM), and X-ray diffraction (XRD) analyses. The UV–vis spectroscopy of AgNPs showed characteristic fingerprint peaks of the green AgNPs predominantly appeared in the range of ∼400–500 nm (Figure 8b). The intensity of the UV–vis spectra of Mo-capped AgNPs was a sharp peak, indicating the high yield of the formed AgNPs. Hosny et al. [26] proved that the AgNPs synthesized by using 20, 30, and 40 g of honey at pH 10 showed sharp absorption peaks spectra at 400 and 460 nm; 415, 435, and 455 nm; and 400, 415, 435, and 450 nm, respectively.

FTIR spectral analysis revealed the functional groups responsible for AgNP synthesis, stabilizing, and capping, with significant common absorption bands at ~3437 cm^−1^, characteristic of υ (O-H) and υ (N-H) vibrational frequencies (Figure 8c). Based on the physical state of the AgNPs and the characteristic features of IR vibrational bands in the spectra, carbonyl was the possible group of the nanoparticle’s reduction and capping. FTIR indicated the importance of molasses ingredients in stabilizing and capping the produced Mo-capped AgNPs within the matrix. XRD analysis showed that the synthesized Mo-capped AgNPs had a spherical shape with distinct diffraction peaks at (32.0), (36.0), and (55.0) angles, which can be indexed by the crystalline planes of AgNPs (Figure 8a). SEM analysis revealed that the produced AgNPs had polymorphic shapes such as rocky, flake type, spherical, and few irregular granulated fused agglomerates (Figure 8d). EDX analysis confirmed the presence of Ag^+^ element strong signals, which confirmed that AgNPs were successfully synthesized. On the other hand, high proportions of Ag^+^ element (78.0%) were detected by EDX analysis (Figure 8e). AgNPs biosynthesized by molasses were examined using TEM, and the obtained micrographs revealed that the average diameter range of the biosynthesized AgNPs was 29–88 nm (Figure 8f).

Changes in NP size may be influenced by the nature and concentration of the polymer (reducing agent) in the organic phase, solvent polarity, and the nature and concentration of the stabilizing agent (surfactants) in the aqueous phase [27]. A change in the reducing agent concentration, pH, temperature, and ratio of reducing agent to AgNO_3_ can have great effects on the physical characteristics of the produced nanoparticles. Logeswari et al. [28] revealed that by changing the reducing agent, different AgNPs size ranges were reported, e.g., 28, 26.5, 65, 22.3, and 28.4 nm, upon using *Ocimum tenuiflorum*, *Syzygium cumini*, *Citrus sinensis*, *Solanum trilobatum*, and *Centella asiatica*, respectively.

## 3. Materials and Methods

### 3.1. Microorganisms

Standard bacterial strains with known biofilm activity were used throughout the present study, namely, *C. albicans* DAY185 and *S. aureus* ATCC 6538, kindly provided by Naval Medical Research Unit (NAMRU) No. 3, Cairo, Egypt.

#### Raw Material

Egyptian sugarcane molasses used in the present study was kindly provided by Naga–Hammady Co. Ltd. (Egypt). The molasses sample was mixed with 74–76% ethanol to precipitate the total sugars present in the sample. The supernatant (containing the polyphenolic compounds) was harvested and concentrated using a rotary evaporator. The concentrated samples were then stored at 5 °C for further investigations [15].

### 3.2. Liquid Chromatography–Mass Spectroscopy Analysis of the Obtained Sugarcane Molasses

Fresh concentrated sample was subjected to liquid chromatography–mass spectroscopy analysis according to Deseo et al. [15] using the Agilent 1290 Infinity UHPLC system (Agilent Technologies, Santa Clara, CA, USA) equipped with the LTQ Orbitrap Velos mass spectrometer (Thermo Fisher Scientific Inc., Waltham, MA, USA). MS analysis was carried out using an electrospray ionization (ESI) interface in negative ion mode. Separation was performed by a C18 column, 150 mm × 2.1 mm 1.9 μ (Thermo Fisher Scientific Inc., Waltham, MA, USA), and the mobile phase was acetonitrile with 0.1% formic acid [15].

### 3.3. Synthesis of AgNPs

A 1 mL volume of 100 mM aqueous solution of AgNO_3_ was added to 2 mL of 10% molasses and stirred well. The mixture was subjected to autoclaving at 15 psi pressure and 121 °C for 20 min [29]. The resulting solution had a dark brown color indicating the formation of silver nanoparticles. The resulting sample was characterized using a UV–vis spectrophotometer and Zetasizer (Malvern Zetasizer Nano ZS, Malvern, UK) to determine the nanoparticles’ size and PDI to confirm AgNP formation and then tested for their antimicrobial activity using the disc diffusion method according to El-Attar et al. [30].

#### 3.3.1. Experimental Design

A multilevel categorical factorial design was used to optimize the response of five input variables and their combinations on the antimicrobial activity (R1), nanoparticles’ size (R2) and polydispersity index (PDI) (R3). The chosen five variables were: molasses (reducing agent) concentration (30% and 40%), pH (6 and 10), ratio of reducing agent AgNO_3_ (3:1 and 4:1), reaction temperature (80 and 120 °C), and reaction time (2 and 3 h) (Table 7) [31]. The reaction mixtures were filtered through 0.22 m Steritop Millipore filters and centrifuged at 12,000 rpm for 15 min to separate the formed AgNPs. To obtain NP powder, each sample was dried and then stored in a refrigerator for further analysis.

##### Box–Behnken Optimization

The optimization tool through response surface methodology (RSM) was employed to study significant level parameters and interactions between variables that influence AgNP synthesis using the Box–Behnken design. The three variables that were considered as the main factors that may potentially affect AgNP synthesis were reducing agent concentration (A) (molasses (20%, 30%, and 40%), pH (B) (6, 8, and 10), and temperature (C) (80, 100, and 120 °C). Table 8 shows the experimental design, consisting of 17 trials, and the independent variables were studied at three different levels: low (−1), medium (0), and high (+1). All experiments were performed in duplicates. The predicted response value Y in each trial of the quadratic model was expressed as:

$$Y={\;\beta }_{0}+\beta_{1}A+\beta_{2}B+\beta_{3}C+\beta_{1}\beta_{1}A^{2}+\beta_{2}\beta_{2}B^{2}+\beta_{3}\beta_{3}C^{2}+\beta_{1}\beta_{2}\mathrm{AB}+\beta_{1}\beta_{3}\mathrm{AC}+\beta_{2}\beta_{3}\mathrm{BC}$$

where, Y is the measured response; β_0_ is the intercept; β_1_, β_2_, and β_3 a_re the linear coefficients; β_1_β_1_, β_2_β_2_, and β_3_β_3 a_re the quadratic coefficients; and β_1_β_2_, β_1_β_3_, and β_2_β_3_ are the interactive coefficients. A, B, and C are the independent variables

Each reaction mixture was centrifuged at 12,000 rpm for 15 min to separate AgNPs, freeze-dried, and analyzed using Zetasizer (Malvern Zetasizer Nano ZS, Malvern, UK) to determine the nanoparticles’ size and PDI. Further analysis was performed by evaluating the nanoparticles’ antimicrobial activity against *C. albicans* DAY185 and *S. aureus* ATCC 6538 through the disc diffusion method [30].

#### 3.3.2. Antimicrobial Activity of the Optimized AgNPs

The antimicrobial activity of optimized AgNPs was further evaluated by MIC, MBC, and microbial lethality curve against *C. albicans* DAY185 and *S. aureus* ATCC 6538 [32]. Transmission electron microscope (TEM) examinations of Mo-AgNP-treated cells of *C. albicans* DAY185 and *S. aureus* ATCC 6538 and consortia of both were also assessed after 12 and 30 h incubation for single and polymicrobial cultures, respectively, using JEM-100 CX Joel at the Electron Microscope Unit, Faculty of Science, Alexandria University, Egypt.

### 3.4. Antibiofilm Activity

#### 3.4.1. Minimal Biofilm Eradication Concentration (MBEC)

Monomicrobial and polymicrobial cultures were grown overnight in liquid broth. The overnight culture (10^8^ CFU/mL) was diluted (1:100) into fresh medium for biofilm assays. Biofilm was allowed to grow for 48 h in a 96-well microtiter plate in the absence and presence of 100 μM of the prepared AgNPs one at a time. The wells were subsequently washed thoroughly with water to remove free-floating and loosely adherent microbial cells, and then the titer plate wells were fixed with 2% sodium acetate and treated with 0.1 mL crystal violet (0.4%) for 15 min [25].

#### 3.4.2. Activity of the Prepared AgNPs against Preformed Biofilm

Biofilms were assessed as previously described with and without AgNPs according to Ansari et al. [25]. Fixed cells were oriented, mounted on the aluminum stubs, and coated with gold before imaging [25]. The topographic features of the biofilms were visualized using SEM (JEOL JSM-6390LV) at the Electron Microscope Unit, Faculty of Science, Alexandria University, Egypt.

### 3.5. Characterization of the Optimized AgNPs

The optimized newly synthesized AgNPs were characterized using a UV–vis spectrophotometer (HITACHI, Model U-2800 spectrophotometer, Omuta-shi, Japan) [30]. The BRUKER FTIR instrument was used for the detection of the FTIR spectra of the prepared AgNPs [32]. X-ray diffraction patterns of the prepared AgNPs were measured using an X-ray powder diffractometer (D8 Advance, BRUKER, Karlsruhe, Germany). SEM (JEOL JSM-6390LV) coupled with the Oxford Instruments EDX was used to examine the surface, size, and shape of the optimized nanoparticles [33]. The ultrastructure, size, and shape of the optimized AgNPs were examined using TEM (JEM-100 CX Joel) [32].

## 4. Conclusions

The bioreduction of silver ions to form silver nanoparticles using a sugarcane process byproduct (molasses) was successfully synthesized and named Mo-capped AgNPs. The synthesized Mo-capped AgNPs had potent antimicrobial activity against *S. aureus* ATCC 6538 and *C. albicans* DAY185. A multilevel categoric factorial design and Box–Behnken optimizations were applied to maximize the antimicrobial activity and enhance the physical characteristics (nanoparticles’ size and PDI) of the synthesized nanoparticles. It was revealed that a molasses concentration of 30%, a pH of 10, and a temperature of 120 °C were the best process conditions. Minimal biofilm eradication concentration (MBEC) of Mo-capped AgNPs and scanning electron microscope study revealed that the prepared AgNPs penetrated the preformed biofilm and eradicated the microbial cells.

## Figures and Tables

**Table 1:** LCMS analysis of the polyphenolic compounds in the obtained sugarcane molasses.

Analyte	RT (min)	Concentration (μg/g)
Chlorogenic acid	8.9	5.91
Caffeic acid	9.7	0.46
Vanillin	12.4	0.21
Orientin	13.5	0.37
Sinapic acid	14.5	0.14
Diosmin	17.1	13.1
Apigenin	18.3	0.01
Diosmetin	20.0	0.24

**Table 2:** Antimicrobial activity of the synthesized AgNPs.

Tested Strains	AgNO_3_		Molasses		Mo-Capped AgNPs	
	IZ (mm)	MIC (µg/mL)	IZ (mm)	MIC (µg/mL)	IZ (mm)	MIC (µg/mL)
*C. albicans* DAY185	R	256.0	R	256.0	13.0 ± 0.79	64.0
*S. aureus* ATCC 6538	15.0 ± 0.58	128.0	R	256.0	19.5 ± 0.91	32.0

IZ: inhibition zone diameter, MIC: minimum inhibitory concentration.

**Table 3:** Mo-capped AgNP synthesis optimization using multilevel factorial design.

Trails	Antimicrobial Activity (Inhibition Zone Diameter, mm)		Physical Characteristics	
	*S. aureus* ATCC 6538	*C. albicans* DAY185	Size (nm)	PDI
1	16.0	9.8	75.0	0.785
2	9.2	8.2	85.0	0.836
3	17.7	11.8	58.2	0.537
4	18.7	10.8	63.7	0.627
5	21.5	8.7	80.0	0.819
6	23.5	10.5	75.0	0.785
7	12.7	13.3	37.4	0.397
8	21.5	10.2	67.7	0.635
9	21.2	11.0	65.2	0.684
10	20.2	12.3	49.3	0.496
11	22.5	9.8	67.7	0.627
12	17.0	9.7	67.7	0.627
13	15.7	9.5	65.2	0.616
14	20.7	8.7	81.2	0.836
15	17.5	12.0	43.1	0.425
16	19.7	10.7	73.1	0.741
17	22.0	7.7	88.0	0.875
18	21.0	8.5	80.0	0.819
19	17.5	8.7	82.0	0.846
20	21.5	8.2	84.0	0.860
21	20.0	11.8	58.2	0.553
22	17.0	10.7	60.1	0.601
23	15.2	12.7	50.1	0.512
24	20.2	12.5	57.3	0.573
25	27.2	16.0	29.0	0.354
26	10.0	12.7	53.5	0.517
27	16.7	11.0	64.3	0.611
28	17.2	8.7	78.0	0.763
29	12.7	10.7	62.4	0.608
30	12.5	9.3	78.0	0.763
31	18.0	11.0	65.4	0.627
32	10.2	11.0	62.4	0.618

**Table 4:** Mo-capped AgNP synthesis optimization using Box–Behnken design.

Trails	Antimicrobial Activity (Inhibition Zone Diameter, mm)		Physical Characteristics	
	*S. aureus* ATCC 6538	*C. albicans* DAY185	Size (nm)	PDI
1	28.0	26.0	52.7	0.467
2	26.0	28.0	81.4	0.724
3	27.0	26.5	75.4	0.634
4	28.0	27.0	80.3	0.712
5	23.0	24.0	38.2	0.375
6	18.0	18.5	88.0	0.776
7	24.5	26.0	35.5	0.362
8	29.0	28.0	39.7	0.383
9	28.0	26.0	47.2	0.434
10	30.0	29.0	29.0	0.356
11	23.6	24.2	50.2	0.467
12	23.7	25.4	42.0	0.410
13	24.5	25.6	61.4	0.567
14	20.1	19.0	52.0	0.534
15	24.0	25.0	46.0	0.434
16	18.3	19.0	72.5	0.612
17	20.0	18.7	65.4	0.578

**Table 5:** MIC and MBC of Mo-capped AgNPs.

Tested Microorganism	Mo-Capped AgNPs (µg/mL)		
	MIC	MBC	MIC Index
*S. aureus* ATCC 6538	4.0	16.0	4.0
*C. albicans* DAY185	16.0	64.0	4.0

MIC: minimum inhibitory concentration, MBC: minimum bactericidal concentration.

**Table 6:** MBEC for the synthesized Mo-capped AgNPs.

Microorganism	MBEC (µg/mL)
*S. aureus* ATCC 6538	16.0
*C. albicans* DAY185	32.0
Consortia *(S. aureus* ATCC 6538 and *C. albicans* DAY185)	32.0

**Table 7:** Multilevel categoric factorial design of Mo-capped AgNP optimization.

Trail/Variable	Reducing Agent Concentration (%)	pH	Ratio (Reducing Agent: AgNO_3_)	Temperature (°C)	Time (h)
1	−1 (30.0)	+1 (10.0)	−1 (3:1)	+1 (120.0)	+1 (3.0)
2	+1 (40.0)	+1 (10.0)	+1 (4:1)	−1 (80.0)	+1 (3.0)
3	−1 (30.0)	−1 (6.0)	+1 (4:1)	+1 (120.0)	+1 (3.0)
4	−1 (30.0)	−1 (6.0)	+1 (4:1)	+1 (120.0)	−1 (2.0)
5	−1 (30.0)	−1 (6.0)	+1 (4:1)	−1 (80.0)	+1 (3.0)
6	−1 (30.0)	+1 (10.0)	+1 (4:1)	+1 (120.0)	−1 (2.0)
7	−1 (30.0)	+1 (10.0)	+1 (4:1)	−1 (80.0)	+1 (3.0)
8	+1 (40.0)	−1 (6.0)	−1 (3:1)	+1 (120.0)	−1 (2.0)
9	+1 (40.0)	+1 (10.0)	−1 (3:1)	+1 (120.0)	+1 (3.0)
10	−1 (30.0)	−1 6.0)	−1 (3:1)	+1 (120.0)	+1 (3.0)
11	−1 (30.0)	−1 (6.0)	−1 (3:1)	+1 (120.0)	−1 (2.0)
12	+1 (40.0)	−1 (6.0)	−1 (3:1)	−1 (80.0)	+1 (3.0)
13	+1 (40.0)	−1 (6.0)	−1 (3:1)	+1 (120.0)	+1 (3.0)
14	−1 (30.0)	+1 (10.0)	−1 (3:1)	−1 (80.0)	+1 (3.0)
15	−1 (30.0)	+1 (10.0)	+1 (4:1)	−1 (80.0)	−1 (2.0)
16	−1 (30.0)	−1 (6.0)	−1 (3:1)	−1 (80.0)	+1 (3.0)
17	+1 (40.0)	+1 (10.0)	+1 (4:1)	−1 (80.0)	−1 (2.0)
18	+1 (40.0)	+1 (10.0)	−1 (3:1)	−1 (80.0)	+1 (3.0)
19	+1 (40.0)	+1 (10.0)	+1 (4:1)	+1 (120.0)	−1 (2.0)
20	+1 (40.0)	+1 (10.0)	−1 (3:1)	+1 (120.0)	−1 (2.0)
21	+1 (40.0)	−1 (6.0)	+1 (4:1)	−1 (80.0)	−1 (2.0)
22	+1 (40.0)	−1 (6.0)	+1 (4:1)	+1 (120.0)	−1 (2.0)
23	−1 (30.0)	−1 (6.0)	+1 (4:1)	−1 (80.0)	−1 (2.0)
24	−1 (30.0)	−1 (6.0)	−1 (3:1)	−1 (80.0)	−1 (2.0)
25	−1 (30.0)	+1 (10.0)	+1 (4:1)	+1 (120.0)	+1 (3.0)
26	+1 (40.0)	−1 (6.0)	+1 (4:1)	−1 (80.0)	+1 (3.0)
27	+1 (40.0)	+1 (10.0)	−1 (3:1)	+1 (120.0)	−1 (2.0)
28	+1 (40.0)	+1 (10.0)	+1 (4:1)	+1 (120.0)	+1 (3.0)
29	+1 (40.0)	−1 (6.0)	+1 (4:1)	+1 (120.0)	+1 (3.0)
30	+1 (40.0)	+1 (10.0)	−1 (3:1)	−1 (80.0)	−1 (2.0)
31	−1 (30.0)	+1 (10.0)	−1 (3:1)	−1 (80.0)	−1 (2.0)
32	+1 (40.0)	−1 (6.0)	−1 (3:1)	−1 (80.0)	−1 (2.0)

**Table 8:** Box–Behnken design of Mo-capped AgNP optimization.

Trial Number	Reducing Agent Concentration (%)	pH	Temperature (°C)
1	0 (30.0)	0 (8.0)	0 (100.0)
2	0 (30.0)	−1 (6.0)	+1 (120.0)
3	+1 (40.0)	0 (8.0)	−1 (80.0)
4	−1 (20.0)	−1 (6.0)	0 (100.0)
5	0 (30.0)	+1 (10.0)	−1 (80.0)
6	+1 (40.0)	−1(6.0)	−1 (80.0)
7	−1 (20.0)	0 (8.0)	−1 (80.0)
8	+1 (40.0)	+1 (10.0)	0 (100.0)
9	+1 (40.0)	−1 (6.0)	0 (100.0)
10	0 (30.0)	+1 (10.0)	+1 (120.0)
11	+1 (40.0)	0 (8.0)	+1 (120.0)
12	−1 (20.0)	+1 (10.0)	+1 (120.0)
13	−1 (20.0)	0 (8.0)	+1 (120.0)
14	1 (40.0)	−1 (6.0)	+1 (120.0)
15	0 (30.0)	−1 (6.0)	0 (100.0)
16	−1 (20.0)	1 (8.0)	−1 (80.0)
17	1 (40.0)	0 (7.0)	0 (100.0)

## Data Availability

Not applicable.

## Supplementary Materials

The following supporting information can be downloaded at: https://www.mdpi.com/article/10.3390/ijms231810243/s1.

## References

[1] Rogers S.A., Bero J.D., Melander C. (2010). Chemical synthesis and biological screening of 2-aminoimidazole-based bacterial and fungal antibiofilm agents. ChemBioChem.

[2] Chen Z., Meng H., Xing G., Chen C., Zhao Y., Jia G., Wang T., Yuan H., Ye C., Zhao F. (2006). Acute toxicological effects of copper nanoparticles in vivo. Toxicol. Lett..

[3] Sheetz T., Vidal J., Pearson T.D., Lozano K. (2005). Nanotechnology: Awareness and societal concerns. Technol. Soc..

[4] Kumar P., Singh P., Kumari K., Mozumdar S., Chandra R. (2011). A green approach for the synthesis of gold nanotriangles using aqueous leaf extract of Callistemon viminalis. Mater. Lett..

[5] Geissel F.J., Platania V., Gogos A., Herrmann I.K., Belibasakis G.N., Chatzinikolaidou M., Sotiriou G.A. (2022). Antibiofilm activity of nanosilver coatings against Staphylococcus aureus. J. Colloid Interface Sci..

[6] Goderecci S.S., Kaiser E., Yanakas M., Norris Z., Scaturro J., Oszust R., Medina C.D., Waechter F., Heon M., Krchnavek R.R. (2017). Silver oxide coatings with high silver-ion elution rates and characterization of bactericidal activity. Molecules.

[7] Thukkaram M., Vaidulych M., Kylián O., Hanus J., Rigole P., Aliakbarshirazi S., Asadian M., Nikiforov A., Van Tongel A., Biederman H. (2020). Investigation of Ag/aC: H nanocomposite coatings on titanium for orthopedic applications. ACS Appl. Mater. Interfaces.

[8] Taglietti A., Arciola C.R., D’Agostino A., Dacarro G., Montanaro L., Campoccia D., Cucca L., Vercellino M., Poggi A., Pallavicini P. (2014). Antibiofilm activity of a monolayer of silver nanoparticles anchored to an amino-silanized glass surface. Biomaterials.

[9] Skandalis N., Dimopoulou A., Georgopoulou A., Gallios N., Papadopoulos D., Tsipas D., Theologidis I., Michailidis N., Chatzinikolaidou M. (2017). The effect of silver nanoparticles size, produced using plant extract from Arbutus unedo, on their antibacterial efficacy. Nanomaterials.

[10] Jamir L., Kumar V., Kaur J., Kumar S., Singh H. (2021). Composition, valorization and therapeutical potential of molasses: A critical review. Environ. Technol. Rev..

[11] Othman A.M., Elsayed M.A., Elshafei A.M., Hassan M.M. (2017). Application of response surface methodology to optimize the extracellular fungal mediated nanosilver green synthesis. J. Genet. Eng. Biotechnol..

[12] Daâssi D., Frikha F., Zouari-Mechichi H., Belbahri L., Woodward S., Mechichi T. (2012). Application of response surface methodology to optimize decolourization of dyes by the laccase-mediator system. J. Environ. Manag..

[13] Poonkuzhali K., Palvannan T. (2011). Thermostabilization of laccase by polysaccharide additives: Enhancement using central composite design of RSM. Carbohydr. Polym..

[14] Lu S.Y., Qian J.Q., Wu Z.G., Ye W.D., Wu G.F., Pan Y.B., Zhang K.Y. (2009). Application of statistical method to evaluate immobilization variables of trypsin entrapped with sol-gel method. J. Biochem. Technol..

[15] Deseo M.A., Elkins A., Rochfort S., Kitchen B. (2020). Antioxidant activity and polyphenol composition of sugarcane molasses extract. Food Chem..

[16] Vilchis-Nestor A.R., Sánchez-Mendieta V., Camacho-López M.A., Gómez-Espinosa R.M., Camacho-López M.A., Arenas-Alatorre J.A. (2008). Solventless synthesis and optical properties of Au and Ag nanoparticles using Camellia sinensis extract. Mater. Lett..

[17] Banerjee P., Satapathy M., Mukhopahayay A., Das P. (2014). Leaf extract mediated green synthesis of silver nanoparticles from widely available Indian plants: Synthesis, characterization, antimicrobial property and toxicity analysis. Bioresour. Bioprocess..

[18] Pourmortazavi S.M., Taghdiri M., Makari V., Rahimi-Nasrabadi M. (2015). Procedure optimization for green synthesis of silver nanoparticles by aqueous extract of Eucalyptus oleosa. Spectrochim. Acta Part A Mol. Biomol. Spectrosc..

[19] Ebrahimzadeh H., Behbahani M., Yamini Y., Adlnasab L., Asgharinezhad A.A. (2013). Optimization of Cu (II)-ion imprinted nanoparticles for trace monitoring of copper in water and fish samples using a Box–Behnken design. React. Funct. Polym..

[20] Nishio E.K., Ribeiro J.M., Oliveira A.G., Andrade C.G., Proni E.A., Kobayashi R.K., Nakazato G. (2016). Antibacterial synergic effect of honey from two stingless bees: Scaptotrigona bipunctata Lepeletier, 1836, and S. postica Latreille, 1807. Sci. Rep..

[21] Haque M.A., Imamura R., Brown G.A., Krishnamurthi V.R., Niyonshuti I.I., Marcelle T., Mathurin L.E., Chen J., Wang Y. (2017). An experiment-based model quantifying antimicrobial activity of silver nanoparticles on Escherichia coli. RSC Adv..

[22] Morones J.R., Elechiguerra J.L., Camacho A., Holt K., Kouri J.B., Ramírez J.T., Yacaman M.J. (2005). The bactericidal effect of silver nanoparticles. Nanotechnology.

[23] Cao C., Zhang T., Yang N., Niu X., Zhou Z., Wang J., Yang D., Chen P., Zhong L., Dong X. (2022). POD Nanozyme optimized by charge separation engineering for light/pH activated bacteria catalytic/photodynamic therapy. Signal Transduct. Target. Ther..

[24] Palanisamy N.K., Ferina N., Amirulhusni A.N., Mohd-Zain Z., Hussaini J., Ping L.J., Durairaj R. (2014). Antibiofilm properties of chemically synthesized silver nanoparticles found against Pseudomonas aeruginosa. J. Nanobiotechnol..

[25] Ansari M.A., Khan H.M., Khan A.A., Cameotra S.S., Alzohairy M.A. (2015). Anti-biofilm efficacy of silver nanoparticles against MRSA and MRSE isolated from wounds in a tertiary care hospital. Indian J. Med. Microbiol..

[26] Hosny A.M., Kashef M.T., Rasmy S.A., Aboul-Magd D.S., El-Bazza Z.E. (2017). Antimicrobial activity of silver nanoparticles synthesized using honey and gamma radiation against silver-resistant bacteria from wounds and burns. Adv. Nat. Sci. Nanosci. Nanotechnol..

[27] Shervani Z., Ikushima Y., Sato M., Kawanami H., Hakuta Y., Yokoyama T., Nagase T., Kuneida H., Aramaki K. (2008). Morphology and size-controlled synthesis of silver nanoparticles in aqueous surfactant polymer solutions. Colloid Polym. Sci..

[28] Logeswari P., Silambarasan S., Abraham J. (2015). Synthesis of silver nanoparticles using plants extract and analysis of their antimicrobial property. J. Saudi Chem. Soc..

[29] Ali Z.A., Yahya R., Sekaran S.D., Puteh R. (2016). Green synthesis of silver nanoparticles using apple extract and its antibacterial properties. Adv. Mater. Sci. Eng..

[30] El-Attar A.A., El-Wakil H.B., Hassanin A.H., Bakr B.A., Almutairi T.M., Hagar M., Elwakil B.H., Olama Z.A. (2022). Silver/Snail Mucous PVA nanofibers: Electrospun synthesis and antibacterial and wound healing activities. Membranes.

[31] Muthukumar M., Mohan D., Rajendran M. (2003). Optimization of mix proportions of mineral aggregates using Box Behnken design of experiments. Cem. Concr. Compos..

[32] Elnaggar Y.S., Elwakil B.H., Elshewemi S.S., El-Naggar M.Y., A Bekhit A., A Olama Z. (2020). Novel Siwa propolis and colistin-integrated chitosan nanoparticles: Elaboration, in vitro and in vivo appraisal. Nanomedicine.

[33] Elnaggar Y.S., Elwakil B.H., Elshewemi S.S., El-Naggar M.Y., A Bekhit A., A Olama Z. (2022). In vivo bio-distribution and acute toxicity evaluation of greenly synthesized ultra-small gold nanoparticles with different biological activities. Sci. Rep..

