# Supplementary material for: Molasses-Silver Nanoparticles: Synthesis, Optimization, Characterization, and Antibiofilm Activity
Source: Int J Mol Sci. 2022 Sep 6;23(18):10243. doi: 10.3390/ijms231810243 (PMC9499626; doi:10.3390/ijms231810243)
Supplement: Supplementary file 1 [file ijms-23-10243-s001.zip › ijms-1875182-supplementary.pdf]

International Journal of
Molecular Sciences

MDPI

Molasses-Silver Nanoparticles: Synthesis, optimization, characterization and antibiofilm activity

***Rabab A. Dorgham*[1], *Mohamed N. Abd Al Moaty*[2], *KhimPhin Chong*[3*], *Zakia A. Olama* [1], *Bassma H. Elwakil*[4*]**

[1]Botany and Microbiology department, Faculty of science, Alexandria University, Egypt.
[2]Chemistry department, Faculty of science, Alexandria University, Egypt.
[3]Faculty of Science and Natural Resources, Universiti Malaysia Sabah, Jalan UMS, Kota Kinabalu, 88400, Sabah, Malaysia
[4]Department of medical laboratory technology, Faculty of Applied Health Sciences Technology, Pharos University in Alexandria, Alexandria, Egypt.

[*]Corresponding author: K-P.C: chongkp@ums.edu.my, B.H.E: bassma.hassan@pua.edu.eg

Table S1. Analysis of variances for response surface second order model in relation to optimization of Mo-capped AgNPs synthesis and statistical analysis by response surface model fitting.

Source	Sum of squares	Degree of freedom (df)	Mean sqaure	F-value	p-value	
**Model**	21.86	3.0	7.29	4.75	0.0298	significant
A-Concentration	4.21	1.0	4.21	2.74	0.1320	
B-pH	17.40	1.0	17.40	11.36	0.0083	
C-Temperature	0.2450	1.0	0.2450	0.1598	0.6986	
**Residual**	13.79	9.0	1.53			
**Cor Total**	35.65	12.0				

Table S2. Regression analysis of ANOVA.

**Std. Dev.**	1.24	**R^2**	0.6131
**Mean**	22.61	**Adjusted R^2**	0.4841
**C.V. %**	5.48	**Predicted R^2**	0.1507
		**Adeq Precision**	6.4072
